# Dispersive Solid Phase Extraction Using Magnetic Nanoparticles Performed in a Narrow-Bored Tube for Extraction of Atorvastatin, Losartan, and Valsartan in Plasma

**DOI:** 10.15171/apb.2019.017

**Published:** 2019-02-21

**Authors:** Mir Ali Farajzadeh, Adeleh Yadeghari, Maryam Abbaspour

**Affiliations:** ^1^Department of Analytical Chemistry, Faculty of Chemistry, University of Tabriz, Tabriz, Iran.; ^2^Engineering Faculty, Near East University, 99138 Nicosia, North Cyprus, Mersin 10, Turkey.

**Keywords:** High–performance liquid chromatography, Magnetic solid phase extraction, Plasma, Atorvastatin, Losartan, Valsartan

## Abstract

***Purpose: ***In this investigation, a new version of magnetic solid phase extraction (MSPE) performed in a narrow–bore tube has been proposed. In this study, hydrophobic octyl (C_8_) functionalized Fe_3_O_4_ magnetic nanoparticles (MNPs) stabilized by SiOH groups (Fe_3_O_4_@SiO_2_@C_8_) are used as magnetic nano–sorbents for the extraction of cardiovascular drugs from human plasma prior to their determination by high performance liquid chromatography–photodiode array detection.

***Methods:*** After precipitation of the plasma proteins, the supernatant is diluted with deionized water and filled into the narrow–bore tube. Then mg–level of the sorbent is added into the tube. The sorbent is dispersed and moved down through the solution instead of passing the solution from the cartridge. Using an external magnet, the collected nano–sorbents at the bottom of the tube are transferred on top of the solution and released to move down through the solution for three times to increase the extraction efficiency.

***Results:*** The linearity of the assay was ranging from 0.4–500 mg mL^-1^. The limits of detection and quantification of the method were obtained in the ranges of 0.05–0.07 and 0.16–0.24 mg L^-1^, respectively. The extraction recoveries were obtained in the range of 31–49%. Intra– and inter–day precisions were calculated and obtained in the ranges of 5–8 and 7%–9% for 0.5 mg L^-1^ of each analyte, and 5–6 and 6%–8% for 2 mg L^-1^ of each analyte, respectively.

***Conclusion:*** The proposed method was successfully used in determination of the studied drugs in patient’s plasmas.

## Introduction

About 30% of the global morbidity and mortality are caused by cardiovascular diseases each year.^1,2^ Hypertension, high cholesterol level or diabetes are some factors that increasing the risk of affection to cardiovascular diseases.^3,4^ All of these factors are caused by stress and unhealthy foods.^2^ The cardiovascular drugs such as atorvastatin, losartan, valsartan, carvedilol, propranolol, verapamil, etc are used for the treatment of the mentioned effected factors.^5^ Atorvastatin belongs to a drug class named statins. It is one of the drugs which is used to help low “bad” cholesterol and fats in blood. It works by reducing the amount of cholesterol made by liver and decreases the risk of heart diseases and helps prevent strokes and heart attacks.^6^ Losartan and valsartan belong to angiotensin-receptor blockers which commonly prescribed for the treatment of hypertension and high blood pressure.^7^

In order to assay different families of drugs used in cardiovascular therapy, various analytical methods have been developed in pharmaceutical and biological samples. In most of them high performance liquid chromatography (HPLC) equipped with ultraviolet,^8^ photo diode array (PDA),^7,9^ fluorescence,^1,10^ or mass spectrometry^11-13^ have been used. Other methods include spectrophotometry,^14,15^ electrochemical method,^16^ gas chromatography,^17^ and capillary electrophoresis.^18^

Sample preparation is an essential step in most analytical procedures which significantly influences the final results of the research, especially in the analysis of biological samples.^19^ Solid phase extraction (SPE) was introduced in 1970s as a powerful clean–up method.^20^ This method has drawbacks such as: clogging of cartridges, being time–consuming, necessity of pump usage, low extraction efficiency resulted from particles aggregation and impossibility treatment of large sample volumes treatment.^21-23^ New SPE methods have attracted special attention in recent years to resolve the mentioned problems. Magnetic solid phase extraction (MSPE) is one of the new SPE procedures which use magnetic sorbents to adsorb analytes. These sorbents can be separated easily using an external magnetic force from an aqueous solution. Then, a suitable solvent is used for desorption of analytes for further determination.^19,24^ Magnetic nanoparticles (MNPs) are a kind of nanoparticles (NPs, 1–100 nm) which present super-paramagnetism and also possess unique reactivity and large specific surface area due to its nano nature.^25^ Different NPs like iron, nickel, cobalt, and their oxides^26^ are used as the core of the sorbents in MSPE that among them Fe_3_O_4_ is often–used NPs.^27,28^ Bare MNPs are seldom directly used in extraction methods because of lack of functional groups to interact with the analytes, aggregation tendency resulting in loss of their magnetism, quick biodegradation, and easily oxidation in air. Thus, MNPs are coated by silane groups for preventing aggregation and oxidation by air and to functionalize of them with different groups which make the sorbents suitable to interact with the analytes.^29-31^ MNPs are functionalized by groups such as C_18_^32^ and C_8_ alkyl chain,^33,34^ phenyl,^35^ graphene,^36^ etc depending on the analytes being extracted.

In this work, an HPLC–PDA method was developed for the simultaneous determination of some cardiovascular drugs (atorvastatin, losartan, and valsartan) in human plasma samples after MSPE performed in a narrow–bore tube using the synthesized Fe_3_O_4_@SiO_2_@C_8_ NPs. In order to obtain suitable sensitivity and separation, factors affecting the chromatographic procedure, such as the composition of mobile phase and its flow rate, and temperature were optimized. Furthermore, the effect of various parameters influencing the extraction efficiency was investigated carefully and the suitable conditions were established. The advantages of the proposed method are short time of chromatographic separation, consuming small amount of biological sample, and eliminating pretreatment of the sorbent which is time–consuming and consumes organic solvent. Also, pumping or suction, and clogging the sorbent observed in the conventional format of SPE were resolved.

## Materials and Methods

### Reagents and standard solutions

All reagents were analytical reagent grade and used without further puriﬁcation. Atorvastatin (as calcium trihydrate), losartan, and valsartan were provided from Darou Pakhsh Pharmaceutical Company (Tehran, Iran). HPLC–grade water, methanol, and acetonitrile were supplied from Caledon (Canada). Deionized water was from Ghazi Pharmaceutical Company (Tabriz, Iran). Phosphoric acid, triethylamine, ammonia, sodium chloride, pyridine, hydrochloric acid, and sodium hydroxide were from Merck (Darmstadt, Germany). Ferric chloride hexahydrate (FeCl_3_.6H_2_O) (99.0%–102.0%) and ferrous sulfate heptahydrate (FeSO_4_.7H_2_O) (99.5%–102.0%) were purchased from Merck (Darmstadt, Germany) and used in the synthesis of Fe_3_O_4_ NPs. Toluene and ethanol were obtained from Fluka (Switzerland). Octyltrimethoxy silane (OTMOS) and tetraethoxy silane (TEOS) with purity of 96% and 98%, respectively, were purchased from Sigma–Aldrich (St. Louis, MO, USA). A magnet (10 × 10 × 0.5 cm) was used in separation of MNPs. Stock solution of each drug (1000 mg L^−1^) was prepared in methanol and stored in a refrigerator at 4°C. A working standard solution (20 mg L^–1^ of each analyte) was prepared in HPLC–grade water by adding appropriate volumes of the stock solutions. This solution was used in preparation of the dilute solutions of the analytes.

### Instrumentation

The size and surface morphology of the MNPs were investigated by scanning electron microscopy (SEM) (Tescan, Czech) with an accelerating voltage of 10.0 kV. Fourier transform infrared (FTIR) spectra were recorded using KBr pellets and FT-IR spectrometer (Bruker, USA) in the range of 400–4000 cm^-1^ with the resolution of 4 cm^-1^. X-ray diffraction (XRD) patterns of all samples were obtained using a Siemens D500 diffractometer (Germany) at room temperature, operating at a voltage of 35 kV. The experiments were done at a scan rate of 1° min^-1^ for a scan range of 5 to 80°.

A Hewlett Packard 1090–II HPLC (Palo Alto, CA, USA) equipped with a 10-μL injection loop, a PDA, and a C_18_ column (25 cm × 4.6 mm i.d.) with a particle size of 5 µm (Alltech, Leonberg, Germany) was used for separation of the analytes. The mobile phase consisted of 0.25%, v/v, triethylamine (adjusted at pH=3.0 by phosphoric acid) and acetonitrile (60:40, v/v) at a flow rate of 1.0 mL min^−1^. The column temperature was thermostated at 40°C. Detection wavelength was set at 205 nm for losartan and valsartan and 200 nm for atorvastatin. The ChemStation software was used for the HPLC system controlling. An LBS2 ultrasonic bath (FALC Instruments, Treviglio, BG, Italy) was used to degas the mobile phase and deionized water used in synthesis of NPs. A Metrohm pH meter model 654 (Herisau, Switzerland), a Labinco vortex model L46 (The Netherlands) and a D–7200 Hettich centrifuge (Kirchlengern, Germany) were used. A VELP Scientiﬁca heating magnetic stirrer (model ARE, Milano, Italy) was used for stirring in MNPs synthesis procedure.

### Synthesis of Fe_3_O_4_@SiO_2_@C_8_ MNPs

This synthesis consists of 3 steps: synthesis of Fe_3_O_4_ NPs, stabilizing of Fe_3_O_4_ NPs using TEOS, and coating of Fe_3_O_4_@SiO_2_ NPs by C_8_ reagent (OTMOS).

Fe_3_O_4_ NPs were prepared by co–precipitation of Fe^3+^ and Fe^2+^ oxides. Initially, analytical grade FeCl_3_.6H_2_O (4.86 g) and FeSO_4_.7H_2_O (3.34 g) were added to 50 mL deionized water placed into the ultrasonic bath for 10 minutes. Then this solution was transferred into a water bath thermostated at a temperature of 100°C and vigorously stirred. After complete dissolving iron salts, 12 mL concentrated ammonia (d = 0.91 mg mL^-1^, 25%, w/v) was added and continued to stir for 2 hours in the mentioned temperature. By adding ammonia, the color of mixture changed to dark black. This solution was cooled and the obtained black NPs were washed until neutral pH with a mixture of ethanol: water (50:50, v/v) in the presence of magnetic field. Finally iron oxide NPs were washed with ethanol and dried in an oven at a temperature of 80°C.

The obtained MNPs from the previous step were suspended into 300 mL mixture of ethanol and deionized water (4:1, v/v) using ultrasonic bath for 15 minutes. Then, 15 mL concentrated ammonia and 6.3 mL TEOS continuously added into the suspension. The mixture was stirred for 12 hours at 40°C and finally the Fe_3_O_4_@SiO_2_ NPs were separated from the solution using the magnet. The obtained MNPs were dried in an oven at a temperature of 60°C.^37^

**Scheme 1:**
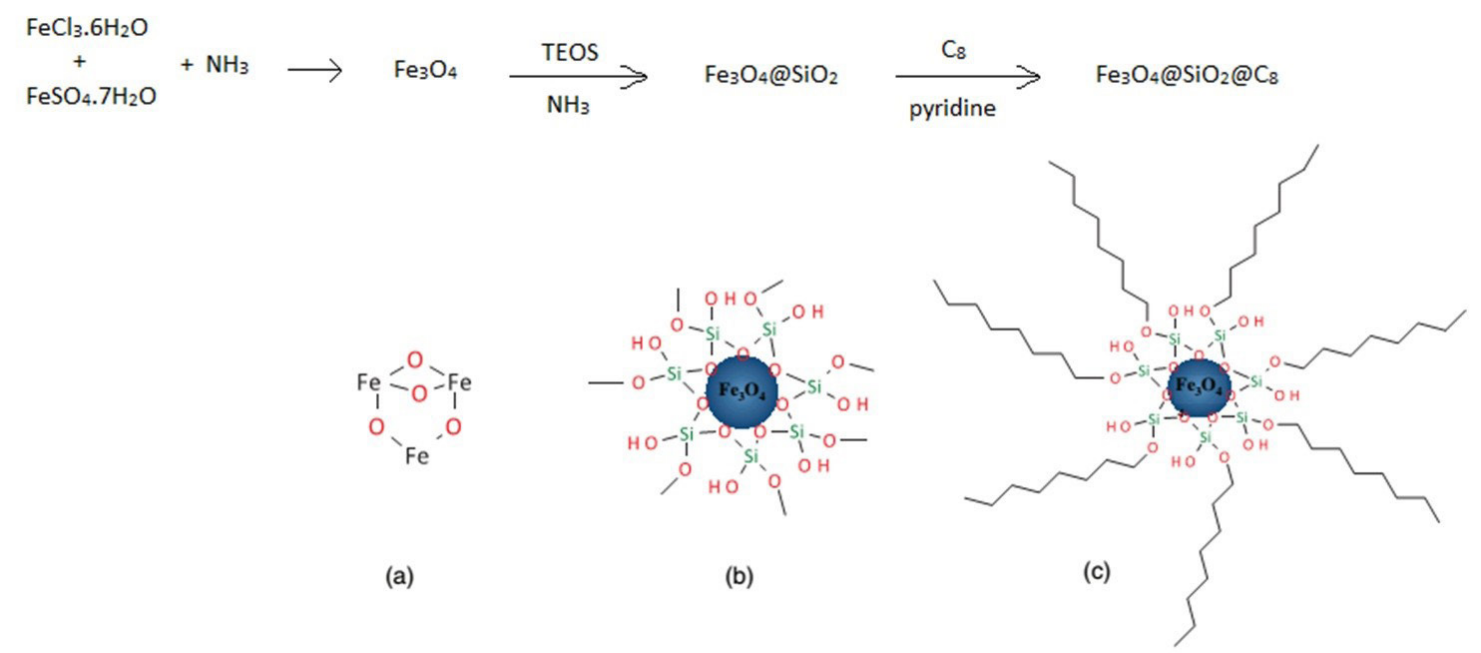
Synthesis of MNPs. Fe_3_O_4_ (a), Fe_3_O_4_@SiO_2_ (b), and Fe_3_O_4_@SiO_2_@C_8_ (c).

Finally for interaction of the MNPs by the analytes, one more step was required too. In this step, the obtained Fe_3_O_4_@SiO_2_ NPs from the previous step were suspended into 50 mL toluene consisted of 2 mL OTMOS by sonicating. After 15 min ultrasonication, 400 µL pyridine as a catalyst was added to the mixture and refluxed for 8 h at a temperature of 120°C. The modified NPs were separated using the magnet and washed by toluene, methanol, and water, respectively, to remove the excess of C_8_ reagent. Finally the produced MNPs were died in an oven at a temperature of 60°C for 1 hour. This step was done according to the procedure reported by Tang et al with partial modification.^29^ It is noted that in this study OTMOS was used instead of phenyltrimethoxy silane. Synthesis steps of the MNPs are shown schematically in Scheme 1.

### Samples

Drugs–free plasma sample (blank plasma) of a healthy human was obtained from the Iranian Blood Transfusion Organization (Tabriz, Iran) and was stored at -20°C before being spiked and used in optimization of the method. Four plasma samples from the patients who received one of the studied drugs (three of them have received atorvastatin, and one of them has received valsartan) and also 3 other plasma samples from the healthy individuals which have not received any of the studied drugs were obtained.

### Microextraction procedure

**Scheme 2:**
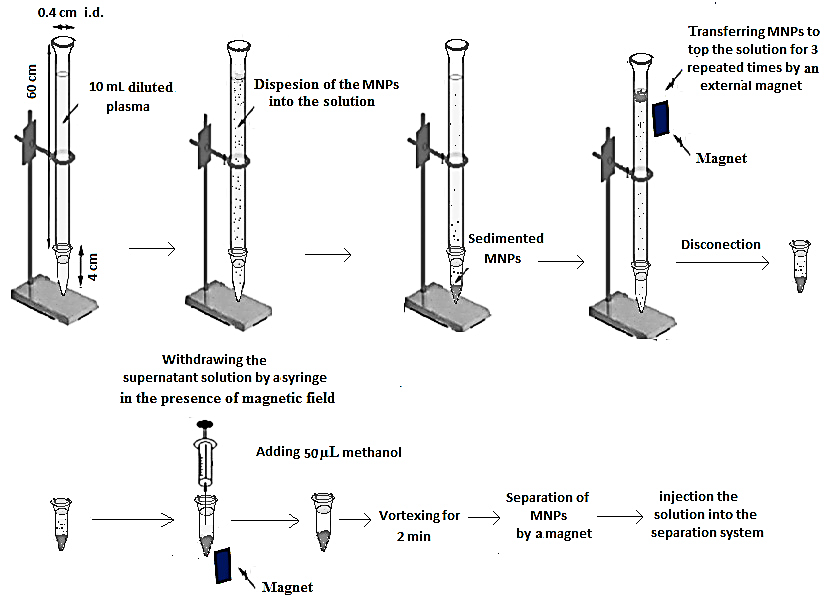
Extraction and preconcentration procedure.

For precipitating the proteins, to 0.5 mL plasma (sample or blank plasma spiked with 1 mg L^-1^ of each analyte), 1 mL methanol was added. After vortexing for 1 minute, the mixture was centrifuged at 7000 rpm for 5 minutes. The supernatant was transferred into a 12–mL test tube and 8.5 mL phosphate buffer (0.1 M, pH = 3) was added to it. Then 2 g NaCl (20%, w/v) was added and vortexed to dissolve. This solution was filled into a home–made set–up. The set–up consisted of a narrow–bore glass tube (60×0.4 cm i.d.) which its head was funnel shaped and its end was connected to a ground glass joint. The tube was connected to a 5–mL ground glass joint test tube with the conical bottom. The volume of this set–up was about 12.5 mL. In the following the synthesized MNPs (20 mg) were added to the solution and the sorbent particles were dispersed into the aqueous solution and moved down through the tube under the gravity force and collected in the bottom of the test tube. Using an external magnet, the collected MNPs at the bottom of the tube were transferred on the top the solution and released to move down through the solution for three repeated times. After this step, the test tube was disconnected. By a 5–mL syringe, the supernatant solution was removed completely in the presence of magnetic field. In the next step, 50 µL methanol as an elution solvent was used for eluting the analytes from the solid sorbent. This mixture was vortexed for 2 minutes and the solution separated from the sorbent by the magnet was injected into the separation system for analysis. Extraction and preconcentration procedure is shown schematically in Scheme 2.

## Results and Discussion

### Characterization of the sorbent

The characterization of MNPs is usually accomplished using techniques such as XRD, SEM, and FTIR.

**Figure 1:**
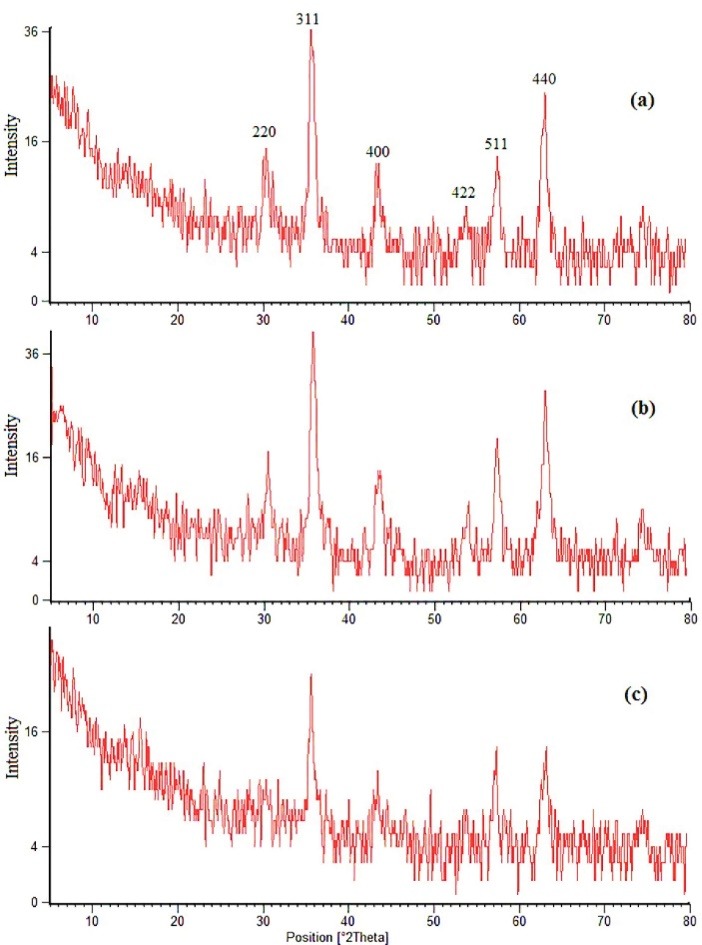
XRD patterns of Fe_3_O_4_ (a), Fe_3_O_4_@SiO_2_ (b), and Fe_3_O_4_@SiO_2_@C_8_ (c).

The XRD patterns of Fe_3_O_4_, Fe_3_O_4_@SiO_2_, and Fe_3_O_4_@SiO_2_@C_8_ are compared in Figure 1 which display several relatively strong reflection peaks in 2θ region of 5-80°. The six characteristic peaks for Fe_3_O_4_ (2*θ*=30.007, 35.601, 43.239, 53.782, 57.372, and 63.058°) are observed which marked by their indices (220, 311, 400, 422, 511, and 440). The peak positions are unchanged upon coating of SiO_2_ and C_8_, indicating that the crystalline structure of the magnetite is essentially maintained.^38^

**Figure 2:**
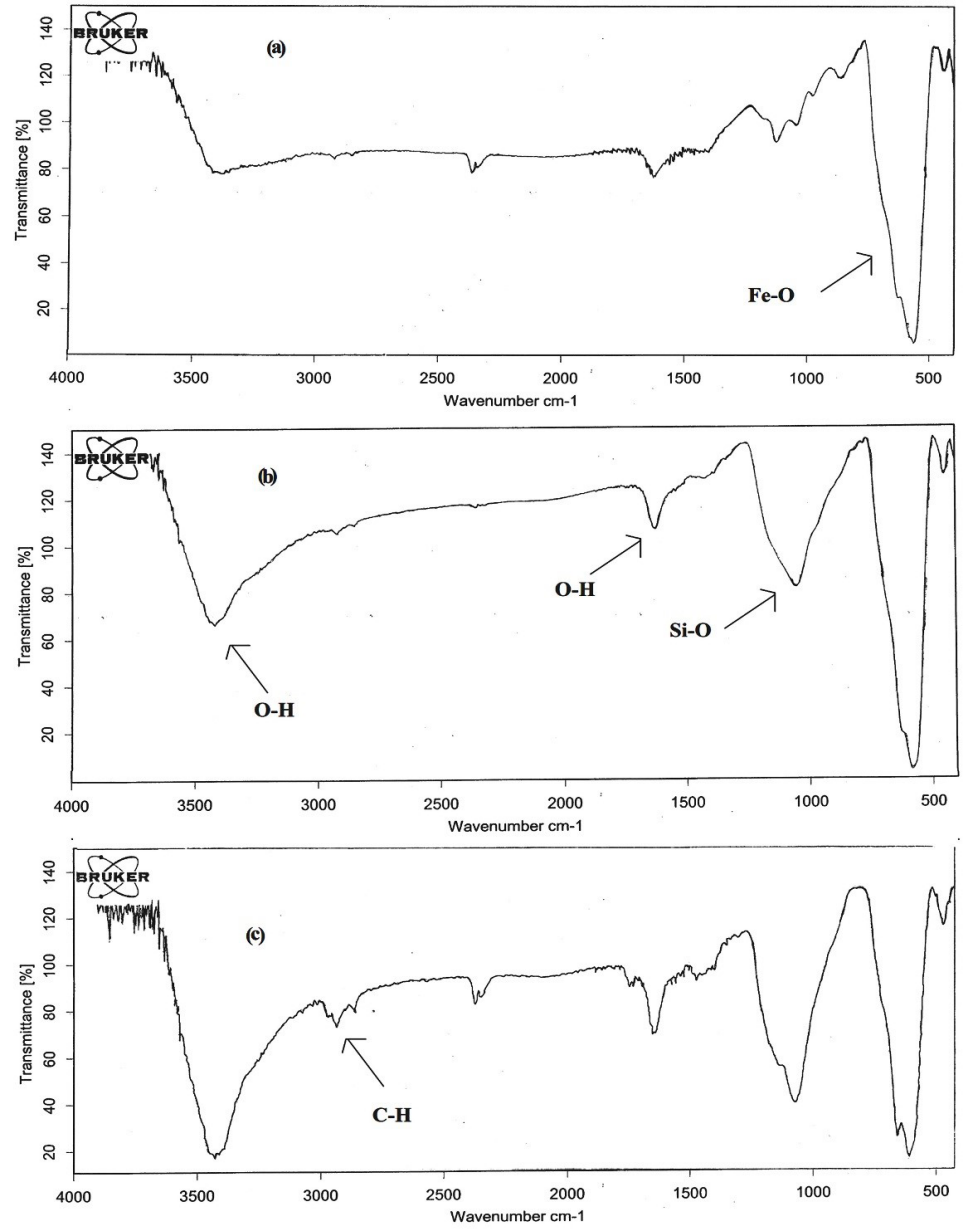
FTIR spectra of Fe_3_O_4_ (a), Fe_3_O_4_@SiO_2_ (b), and Fe_3_O_4_@SiO_2_@C_8_ (c).

Figure 2 shows the FT-IR spectra of Fe_3_O_4_, Fe_3_O_4_@SiO_2_, and Fe_3_O_4_@SiO_2_@C_8_. The strong peak around 570 cm^-1^ is attributed to the characteristic absorbance band of Fe-O in Fe_3_O_4_ (Figure 2a). In comparison to Figure 2a, Figure 2b has new peaks at 1052 cm^-1^ (Si-O in SiO_2_ stretching vibration) and 1626 cm^-1^ and 3419 cm^-1^ which are related to O-H. A peak at 2923 cm^-1^ in Figure 2c is related to alkane C-H that proves the sticking of C_8_ to Fe_3_O_4_@SiO_2_.

**Figure 3:**
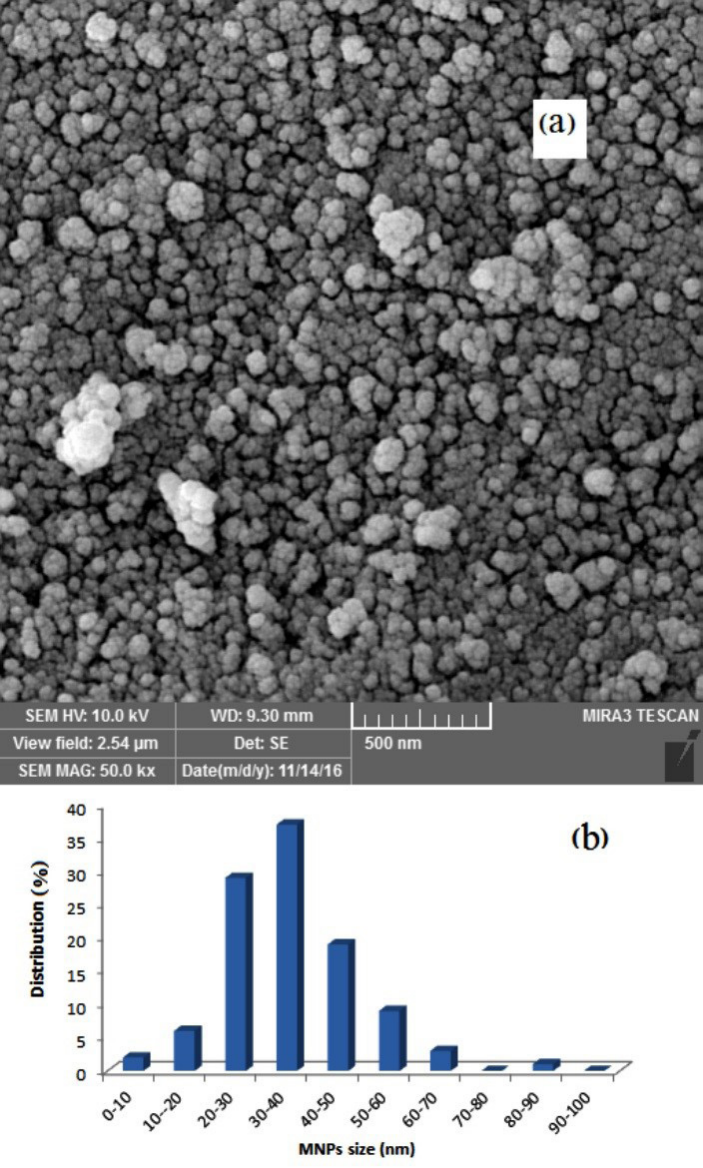
SEM image of Fe_3_O_4_@SiO_2_@C_8_ (a), and size distribution (b).

The morphological characteristics (size and shape) of the magnetic Fe_3_O_4_@SiO_2_@C_8_ indicated by SEM are shown in Figure 3 which spherical shape of the MNPs can be observed with an average diameter of 35 nm. The most distribution of MNPs based on Figure 3b is in the range of 30-40 nm. Also, the SEM micrograph shows some agglomerates.

### Optimization of the extraction conditions

In order to find the best conditions for the extraction and preconcentration of the studied drugs, different parameters affecting the extraction efficiency were investigated in details. These parameters were amount of the sorbent, ionic strength of the solution, kind and volume of the elution solvent, extraction numbers, and ph.

#### Study of ionic strength

**Figure 4:**
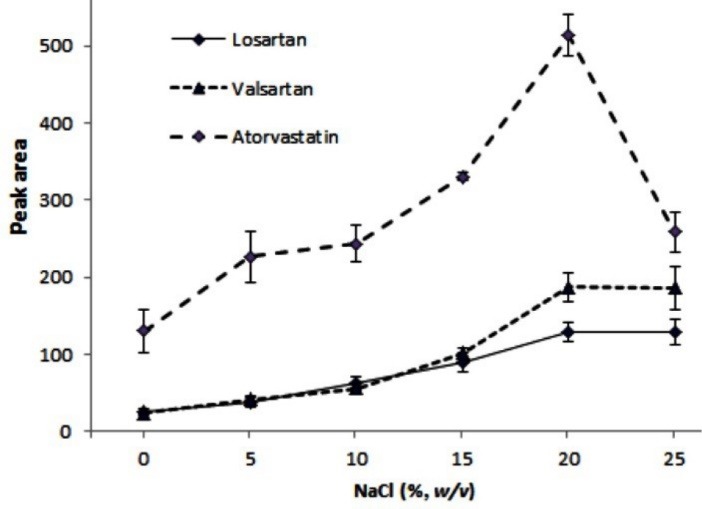
Study of ionic strength: Extraction conditions: sample, 0.5 mL drug–free plasma was spiked with 1 mg L^–1^ of each analyte. After precipitating its proteins by 1 mL methanol, the supernatant was diluted with phosphate buffer (0.1 M, pH=3) to make its volume 10 mL; extraction numbers, 3; elution solvent, 100 µL methanol; and vortex time, 2 min; sorbent amount: 20 mg. The error bars indicate standard deviation of 3 repeated determinations.

The effect of ionic strength on efficiency of the extraction method can be explained in 2 ways: (*a*) viscosity of the aqueous solution increases in high concentration of salt which, in turns, results in the decreased diffusion coefficients of the analytes and the reduced extraction efficiency, and (*b*) by adding a salt, solubility of the analytes in the aqueous phase decreases because of increasing the polarity of the aqueous solution, so extraction efficiency can be Improved. This phenomenon is called “salting out” effect. The ionic strength effect on the extraction efficiency was investigated in the presence of 0%, 5%, 10%, 15%, 20%, and 25%, w/v, NaCl. By increasing the salt concentration, the analytical signals increased up to 20%, w/v, because of salting out effect, and then decreased or remained constant at 25%, w/v, as a result of aqueous solution viscosity increasing. With regard to these results (Figure 4), the further experiments were performed in the presence of 20%, w/v, NaCl.

#### Study of aqueous solution pH

**Figure 5:**
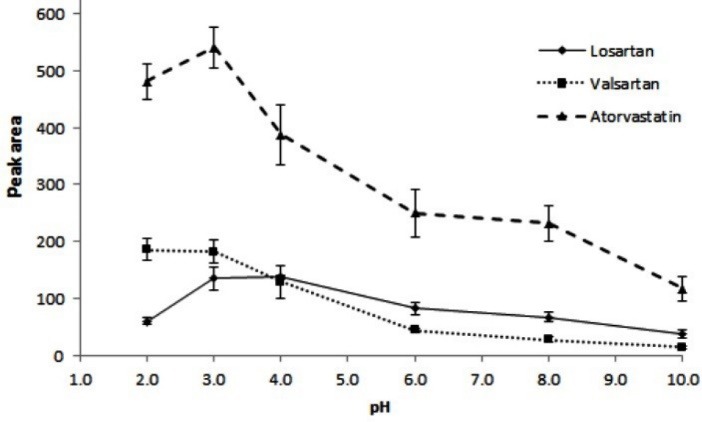
Influence of pH on the extraction efficiency of the method. Extraction conditions: NaCl concentration: 20%, w/v, and 8.5 mL Robinson buffer (C=0.12 M) was used instead of phosphate buffer. Other extraction conditions are the same as used in Figure 4.

For adsorption of the studied analytes onto the sorbent containing C_8_ groups (non–polar groups), the analytes should be in molecular forms. For finding the optimum pH in extraction of the analytes, different pH values ranged in 2-10 were tested. In each case, 8.5 mL of a universal buffer (Robinson buffer, 0.12 M) in the mentioned pHs was used. The results in Figure 5 show that the high extraction efficiency of the studied analytes is obtained at pH 3. Regarding to pK_a_ of the studied drugs (valsartan, 4.37; losartan, 5.5; and atorvastatin, 4.3),^39^ extraction of the analytes in acidic pHs (lower than 4) is logical. Decrease of peak areas at pH = 2 may be related to the sorbent decomposition. So, phosphate buffer (C=0.1 M) with a pH of 3 was used for the next steps.

#### Optimization of the sorbent amount

**Figure 6:**
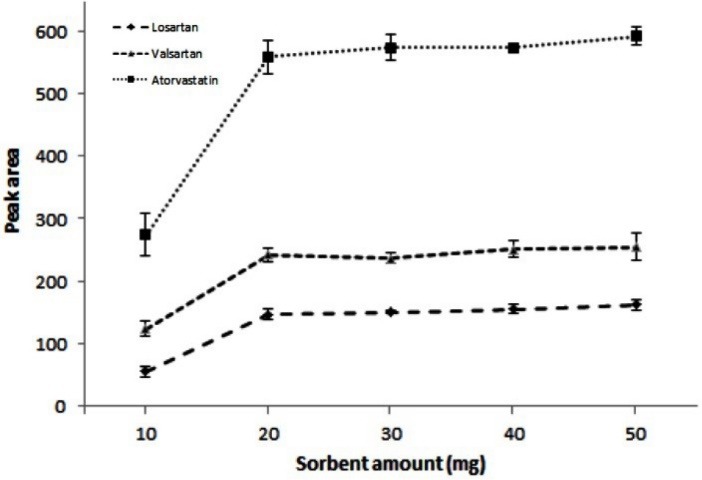
Effect of the sorbent amount on the method efficiency. Extraction conditions are the same as used in Figure 4 except phosphate buffer (0.1 M, pH=3) was used instead of Robinson buffer.

Different amounts of the sorbent (10, 20, 30, 40, and 50 mg) were tested to optimize the sorbent amount. The results in Figure 6 indicate that peak areas increase up to 20 mg and then reach the constant amounts. Therefore 20 mg was selected as the optimal amount of the sorbent for the subsequent experiments.

#### Study of extraction numbers

**Figure 7:**
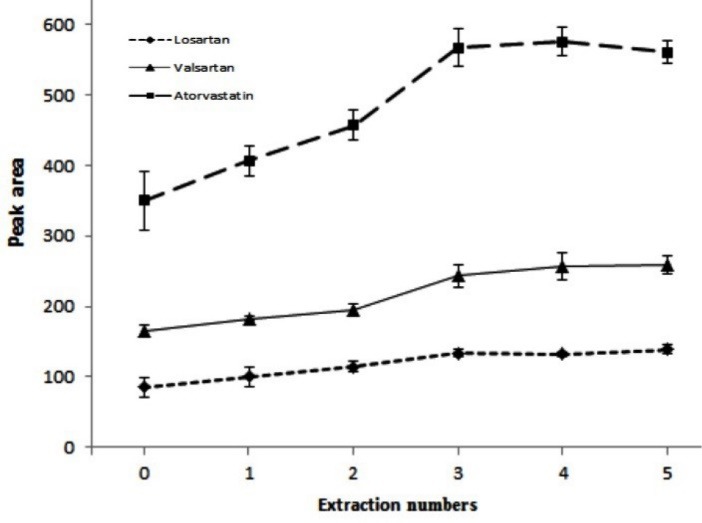
Optimization of extraction numbers. Extraction conditions are the same as used in Figure 4, except 20 mg of the sorbent was used.

After adding the sorbent into the solution placed in the extraction device, it moves through the tube based on gravity force. Using an external magnet, the collected sorbent at the bottom of the tube was transferred on top of the solution and let to move again through the tube. In this study, the number of times that the sorbent moved by the magnet on top of the solution is called as the extraction numbers. It is reasonable that high extraction numbers lead to high analytical signals. For finding the optimal extraction numbers, 0, 1, 2, 3, 4, and 5 times were tested. Figure 7 indicates that the analytical signals increase by increasing extraction numbers up to 3 and then remain constant. Hence 3 times was selected as the optimal numbers for the extraction of the studied drugs.

#### Selection of elution solvent kind and its volume

**Figure 8:**
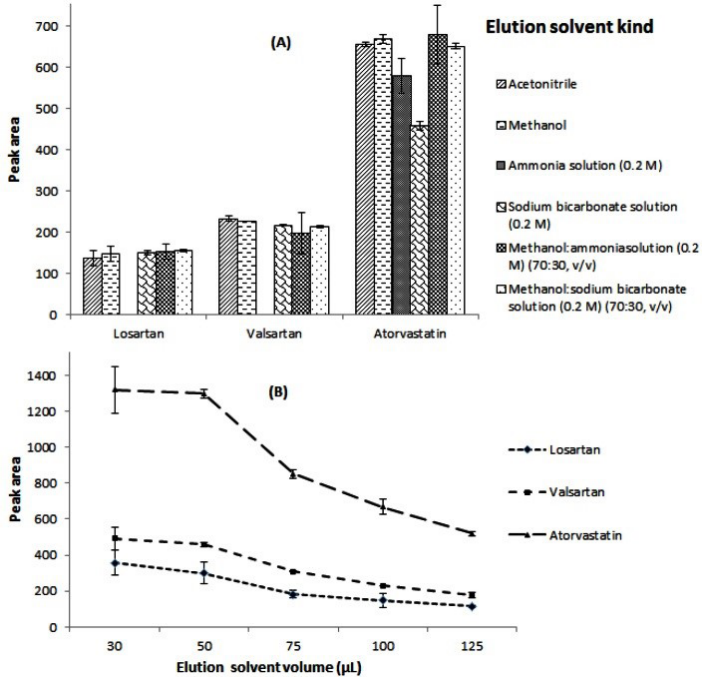
Selection of elution solvent type (A), and its volume (B). Extraction conditions are the same as used in Figure 4, except extraction numbers of 3 were used.

Finding a suitable solvent for elution of the analytes sorbed onto the sorbent is an important step in the proposed method. For investigating this parameter, different elution solvents including methanol, acetonitrile, sodium bicarbonate solution (0.2 M), ammonia solution (0.2 M), and mixtures of methanol: 0.2 M ammonia solution (70:30, v/v), and methanol: 0.2 M sodium bicarbonate solution (70:30, v/v) were tested. Comparison of the peak areas obtained using different solvents indicates that methanol, acetonitrile, and mixture of methanol: sodium bicarbonate solution gave the highest extraction efficiencies for the target analytes among the selected solvents (Figure 8A). However methanol was chosen for the further studies.

Also, volume of the elution solvent (methanol) can be another parameter that should be considered. For optimization of this parameter 30, 50, 75, 100, and 125 µL methanol were tested. According to the obtained data in Figure 8B, in the case of 30 µL the highest analytical signals are achieved but repeatability of the method is not satisfied. This can be concluded from the error bars. Also, separation of the sorbent from the solution was difficult. So, 50 µL was chosen as the optimal volume of the elution solvent.

#### Optimization of vortex time

In this study, different vortexing times (0.5, 1.0, 1.5, 2.0, and 2.5 minutes) were investigated under a constant rotational speed for dissolving the dried analytes in methanol. The extraction efficiency increased up to 1.5 minutes and after that, no considerable change was observed. Therefore, 2 minutes as the vortexing time was selected for the further studies.

#### Investigation of other drugs interferences

An interesting aspect of each analytical method is determination of trace concentration of analytes in the presence of the other species that their presence in real samples is plausible. So the effect of different drugs on the relative recoveries of the studied analytes was investigated by spiking 2 mg L^-1^ of each amiodarone, propranolol, verapamil, carvedilol, atenolol, acetaminophen, ibuprofen, diclofenac, and amoxicillin into the blank plasma fortified with 500 µg L^-1^ of each studied drug. The obtained results are gathered in Table 1. As it can be seen the obtained relative recovery are between 93% and 104%. These results indicate that the proposed method can be used for the determination of the analytes in plasma in the presence of other selected drugs.

**Table 1:** Interferences study

**Drug**	**Relative recovery (%) ± standard deviation (n=3)**		
	**Losartan**	**Valsartan**	**Atorvastatin**
Amiodarone	94 ± 2	97 ± 4	96 ± 3
Propranolol	97 ± 3	97 ± 2	95 ± 3
Verapamil	102 ± 3	104 ± 5	96 ± 2
Carvedilol	96 ± 4	95 ± 3	97 ± 1
Atenolol	96 ± 3	93 ±4	95 ± 3
Acetaminophen	96 ± 2	97 ± 1	98 ± 3
Ibuprofen	97 ± 4	94 ± 4	93 ± 3
Diclofenac	99 ± 4	97 ± 3	97 ± 2
Amoxicillin	101 ± 2	98 ± 3	99 ± 2

#### Quantitative features of the method

To evaluate the proposed method, linear range of the calibration graph, coefficient of determination (R^2^), limit of detection (LOD), limit of quantification (LOQ), relative standard deviation (RSD), and extraction recovery (ER) were investigated under the optimized conditions. The results are listed in Table 2. The linearity of the assay was evaluated by constructing the calibration curves with different concentrations of the analytes (n=11) ranging from 0.4–500 mg mL^-1^. The R^2^ for losartan, valsartan, and atorvastatin were 0.997, 0.998, and 0.998, respectively. The LODs and LOQs of the method were estimated based on signal intensity 3 and 10 times more than baseline noise (S/N=3) and (S/N=10), respectively. Based on these definitions, the LODs and LOQs were obtained in the ranges of 0.05–0.07 and 0.16–0.24 mg L^-1^, respectively. It is noted that therapeutic plasma concentrations of atorvastatin, losartan, and valsartan are 0.2–1.2, 0.8–6, and 0.032–0.103 mg L^−1^, respectively which are higher than the method LODs, except for atorvastatin in low range.^9,40^ The ERs were obtained in the range of 31%-49%, which are acceptable in plasma matrix. For assessment of the method precision, replicated analysis of the analytes at two concentrations (0.5 and 2 mg L^-1^ of each analyte in plasma) were done within a same day (n=6) and different days (n=3). Intra– and inter–day RSDs were calculated and obtained in the ranges of 5–8 and 7%–9% for 0.5 mg L^-1^, and 5–6 and 6%–8% for 2 mg L^-1^, respectively.

**Table 2:** Analytical features of the proposed MSPE–HPLC–PDA method in human plasma

**Analytes**	**LOD** ^a^ **(mg L** ^−1^ **)**	**LOQ** ^b^ **(mg L** ^−1^ **)**	**LR** ^c^ **(mg L** ^−1^ **)**	**R** ^2d^	**Calibration curve equation**	**ER±SD** ^e^	**RSD %** ^f^		**RSD %** ^g^	
							**Intra–day** **(n=6)**	**Inter–day** **(n=3)**	**Intra–day** **(n=6)**	**Inter–day** **(n=3)**
Losartan	0.07	0.24	0.4–500	0.997	Area = 128.38C+39.362	31 ± 5	8	9	6	8
Valsartan	0.07	0.23	0.4–500	0.998	Area = 461.18C+54.632	38 ± 5	7	7	6	7
Atorvastatin	0.05	0.16	0.4–500	0.998	Area = 786.35C+54.631	49 ± 4	5	7	5	6

^a^Limit of detection, (S/N = 3), ^b^Limit of quantification, (S/N = 10), ^c^ Linear range, ^d^ Coefficient of determination, ^e^Extraction recovery ± standard deviation (n= 3), ^f^Relative standard deviation (C = 0.5 mg L^-1^ of each analyte), ^g^Relative standard deviation (C = 2 mg L^-1^ of each analyte).

#### Plasma samples analysis

**Figure 9:**
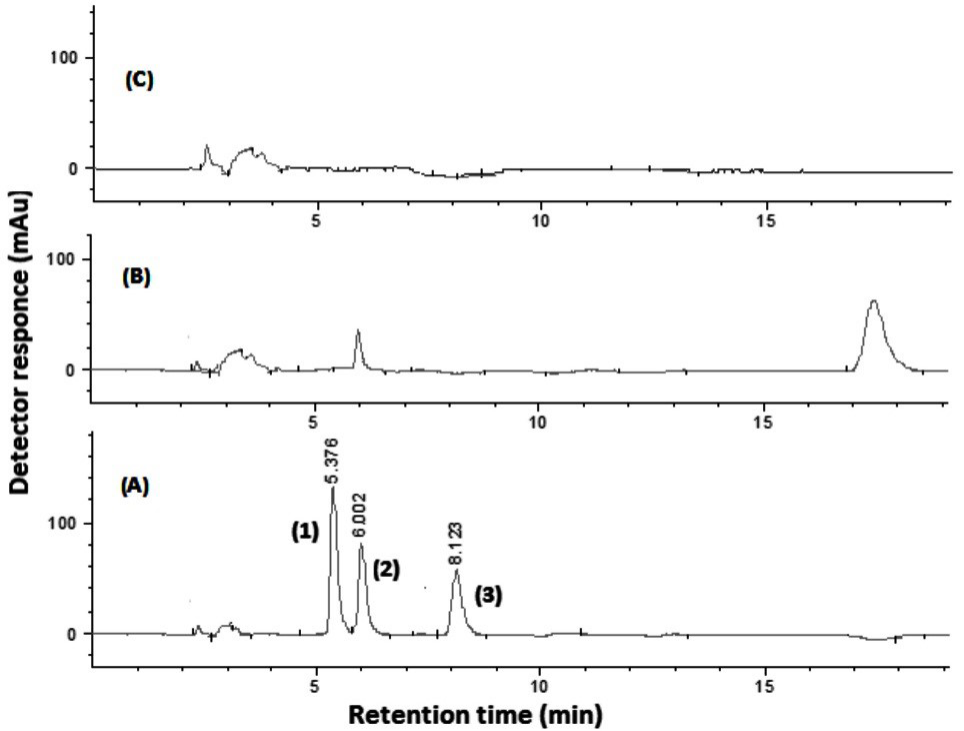
HPLC–PDA chromatograms of: (A) standard solution (20 mg L^-1^ of each analyte), (B) plasma sample of patient treated with valsartan, and (C) drug–free plasma of a healthy person. In all cases, except (A) the proposed method was performed and the enriched analytes were injected into the separation system. In chromatogram (A) direct injection was carried out. Detection wavelength was 205 nm. Peaks identification: (1) losartan, (2) valsartan, and (3) atorvastatin.

The proposed method was applied in the analysis of plasma samples of the patients under cardiovascular treatment (four plasma samples from the patients who received atorvastatin and valsartan orally) for demonstrating the applicability of the method. Also three other plasma samples obtained from healthy volunteers received none of the studied drugs were analyzed by the method. In the cases of drug–free plasma samples and plasma of the patient received atorvastatin, no peak was found in the retention times of the analytes. In patient sample, the found concentration was calculated 0.72 mg L^−1^ for valsartan sample. Typical HPLC–PDA chromatograms corresponding to a standard solution of the analytes (20 mg L^−1^ of each), drug–free plasma sample, and plasmas of the patients under treatment with valsartan are shown in Figure 9.

## Conclusion

In this study, a new version of MSPE performed in a narrow–bore tube was proposed for the simultaneous determination of atorvastatin, losartan, and valsartan (cardiovascular drugs) in human plasma prior to their HPLC–PDA analysis. The major advantages of the method are its short separation time (<8 minutes), small quantity of the required solvents, and avoiding cartridge format. There are other exclusive advantages in this method compared to traditional SPE method such as elimination of vacuum pump and conditioning step of the sorbent. Also, the analytical features of the method show satisfactory linearity, precision, LOD, and ER under the optimized conditions.

## Acknowledgments

The authors thank the Research Council of University of Tabriz and Iran National Science Foundation (Science Research No. PHD 96000434) for the financial support.

## Conflict of Interest

There is no conﬂict of interest for this work; all authors are in agreement with the submission of this research paper to this journal.

## Ethical Issues

Not applicable.
